# Labor migration is associated with lower rates of underweight and higher rates of obesity among left-behind wives in rural Bangladesh: a cross-sectional study

**DOI:** 10.1186/s12992-021-00712-5

**Published:** 2021-07-18

**Authors:** Kristin K. Sznajder, Katherine Wander, Siobhan Mattison, Elizabeth Medina-Romero, Nurul Alam, Rubhana Raqib, Anjan Kumar, Farjana Haque, Tami Blumenfield, Mary K. Shenk

**Affiliations:** 1 Pennsylvania State University College of Medicine Department of Public Health Sciences, Hershey, USA; 2 Binghamton University State University of New York Department of Anthropology, Binghamton, USA; 3 University of New Mexico Department of Anthropology, Albuquerque, USA; 4 icddr, b, Dhaka, Bangladesh; 5 Pennsylvania State University Department of Anthropology, State College, USA

**Keywords:** Nutrition transition, Migration, Rural wives left behind, Chronic disease, Bangladesh

## Abstract

**Background:**

Among Bangladeshi men, international labor migration has increased ten-fold since 1990 and rural to urban labor migration rates have steadily increased. Labor migration of husbands has increased household wealth and redefined women’s roles, which have both positively and negatively impacted the health of wives “left behind”. We examined the direct and indirect effects of husband labor migration on chronic disease indicators and outcomes among wives of labor migrants.

**Methods:**

We collected survey, anthropometric, and biomarker data from a random sample of women in Matlab, Bangladesh, in 2018. We assessed associations between husband’s migration and indicators of adiposity and chronic disease. We used structural equation modeling to assess the direct effect of labor migration on chronic disease, undernutrition, and adiposity, and the mediating roles of income, food security, and proportion of food purchased from the bazaar. Qualitative interviews and participant observation were used to help provide context for the associations we found in our quantitative results.

**Findings:**

Among study participants, 9.0% were underweight, 50.9% were iron deficient, 48.3% were anemic, 39.6% were obese, 27.3% had a waist circumference over 35 in., 33.1% had a high whole-body fat percentage, 32.8% were diabetic, and 32.9% had hypertension. Slightly more women in the sample (55.3%) had a husband who never migrated than had a husband who had ever migrated (44.9%). Of those whose husband had ever migrated, 25.8% had a husband who was a current international migrant. Wives of migrants were less likely to be underweight, and more likely to have indicators of excess adiposity, than wives of non-migrants. Protection against undernutrition was attributable primarily to increased food security among wives of migrants, while increased adiposity was attributable primarily to purchasing a higher proportion of food from the bazaar; however, there was a separate path through income, which qualitative findings suggest may be related to reduced physical activity.

**Conclusions:**

Labor migration, and particularly international labor migration, intensifies the nutrition transition in Bangladesh through increasing wealth, changing how foods are purchased, and reducing physical activity, which both decreases risk for undernutrition and increases risk for excess adiposity.

**Supplementary Information:**

The online version contains supplementary material available at 10.1186/s12992-021-00712-5.

## Introduction

Rural Bangladesh is currently undergoing a nutrition transition, defined by changes in diet and physical activity patterns associated with increasing rates of morbidity and mortality due to chronic disease [1, 2]. The nutrition transition hypothesis posits that populations experience similar changes in subsistence, diet, and physical activity with increasing economic development and globalization. This is thought to present in five stages with the first three stages being collection of food, nutritional stress due to famine, and the end of famine. The last two stages include (a) diets high in fat and sugar alongside a sedentary lifestyle, which is presently occurring in much of the less developed world, and (b) improved diet to include nutritious foods alongside increased physically activity, which is presently occurring in high-income countries [3]. Epidemiologic transitions are often linked with nutrition transitions, as diets high in fat and sugar, especially co-occurring with low physical activity, increase rates of overweight and obesity in previously lean or underweight populations [4] and contribute to risk for chronic disease [5] including for hypertension and diabetes [6, 7]. The last major famine occurred in Bangladesh in the mid-1970s, and the transition towards less healthy diets and lower levels of physical activity is presently occurring in Bangladesh. Evidence exists for a nutrition transition in Bangladesh with increasing prevalence of overweight and obesity in urban and rural areas, alongside a decreasing prevalence of underweight and micronutrient deficiency, including iron deficiency and anemia [2, 8]. Consistent with an epidemiologic transition, mortality from chronic diseases such as cardiovascular disease, hypertension, and diabetes has also increased in Bangladesh over the past several decades [9–11].

Increasing rates of labor migration have dramatically redefined family structures in rural Bangladesh, leaving wives with increased purchasing power, which has potential implications for nutrition [12]. A large fraction of labor migrants from rural Bangladesh are men whose wives and children remain in the village [13]. The number of rural Bangladeshi men moving to urban areas within Bangladesh and abroad to pursue economic opportunities is expected to continue to increase, as persistent population growth and climate change yield smaller landholdings and poorer farming conditions, which push men out of rural communities, while urban employment opportunities continue to surge, with economic development and globalization pulling men toward large population centers [14, 15]. Wealth from remittances in rural areas of Bangladesh is growing [13, 16]. Because labor migration generally results in greater wealth and economic stability for families ‘left behind’, families pursue such opportunities despite expectations for long-term family separation [17].

Significant variation exists in patterns of labor migration and remittance. Some migrants move abroad (e.g. Oman, Qatar, Singapore, and United Arab Emirates) to pursue unskilled or skilled labor at relatively high wages, while others with more limited resources move to pursue unskilled and skilled labor jobs within Bangladesh [18, 19]. A large fraction of labor migrants send regular remittances to their families in the village, allowing significant investment in household maintenance, healthcare for their kin, and the education and marriages of their children [5]. The experiences of the families of men who migrate for work abroad versus men who migrate to other parts of Bangladesh may differ, as international labor migration tends to lead to higher earnings and larger flows of remittances [20]. Higher remittances have been found to increase financial savings, allowing families to accumulate wealth [20].

Increased wealth through remittances may improve migrants’ families’ diets, reducing undernutrition. Yet increased wealth may also contribute to obesogenic and diabetogenic diets, increasing risk for cardiovascular disease, hypertension, and diabetes [21]. Additionally, with rising socio-economic status (SES), activity levels have been shown to decrease, particularly among women, which could also exacerbate risks for chronic disease [22, 23]. It is possible that women with migrant husbands are more likely to use betelnut compared with women whose husbands do not migrate. Betelnut is a widely used addictive substance among Bangladeshi adults and can increase all cause and cancer-related mortality and the risk for diabetes and hypertension [24–26]. Wives ‘left behind’ may be adversely affected by psychosocial stress from the loss of their husband’s social support and/or household labor or altered family dynamics [27, 28]. Psychological stress has been linked with several chronic diseases, including hypertension [29], diabetes [30], obesity [31], and anemia [32]. Psychosocial stress may be exacerbated if husbands are gone for longer periods, as is generally the case for international labor migrants from Bangladesh, who often remain in their destination country for years at a time. By contrast, domestic migrants may remain in their destinations for only days or weeks before returning to visit their families in the village.

We assessed associations between (a) having a labor migrant husband and (b) having a husband who migrated internationally, and indicators of chronic disease among a sample of married rural Bangladeshi women. We hypothesized that wives ‘left-behind’ by migrant husbands will experience an increase in indicators of adiposity and chronic disease, and a reduction in underweight and micronutrient deficiencies. We explored the extent to which marriage to a labor migrant and specifically to an international labor migrant was independently associated with chronic disease outcomes, indicating an effect of psychosocial stress, and the extent to which predicted associations were mediated by increased household monthly income, food security, and/or purchasing (rather than producing) food.

## Methods

Survey, anthropometric, and biomarker data were collected in 2018 from a random sample of women living in Matlab, Bangladesh. The sample was drawn from a full population roster kept by our collaborating organization icddr,b (formerly known as the International Centre for Diarrhoeal Disease Research, Bangladesh) as part of their ongoing Health and Demographic Surveillance System (HDSS) study area which has been in operation since the 1960s [33]. Women were eligible for inclusion if they were between 20 and 65 years of age in 2010, when the original wave of study data were collected, and remained in the study area in 2018. Each wave of data collection (2010 and 2018), began with a pretesting phase, during which survey items were pretested and (in 2018) acceptability of health screening tests was evaluated. Survey items and health tests were modified as needed for clarity and acceptability. Pretesting was conducted among participants not included in the main sample. After the pretesting phase was complete, data were collected from the main sample.

944 women were originally interviewed in 2010, and 765 of these women remained in the study area and were re-interviewed in 2018; attrition was primarily related to families moving out of the study area (*N* = 154), with a limited number of cases attributable to death (*N* = 4), disability (*N* = 14), refusal (N = 4, typically due to illness or pressing obligations), or prolonged travel outside of the study area (*N* = 3). Data in this paper are exclusively from the women followed up in 2018. For the current analysis, unmarried women and women who were pregnant at the time when health data were collected in 2018 were excluded.

### Socio-economic characteristics

A questionnaire was used to collected participants’ age or year of birth from which age was calculated (years), education (years), religion (Islam or Hinduism), monthly household income (taka), food security, proportion of food procured from the bazaar (as opposed to food produced by the family), and current betelnut use (yes/no). Food procured from the bazaar was considered as a dichotomous variable for all food consumed purchased from the bazaar, compared to some or no food consumed purchased from the bazaar. Participants were considered food secure if they reported always having enough food, compared to having enough food but of poor quality, or not having enough food on a monthly, weekly, or daily basis. Household income was right skewed, so we used the natural logarithm of income for analysis.

### Chronic disease outcomes

Anthropometry was performed in participants’ households at the time of questionnaire administration. Standing height was estimated with a portable stadiometer (the model used was a Seca 213). Weight (kg) and whole body fat percentage (BF%) were estimated with a portable digital scale with bioelectrical impedance (the model used was a Tanita BC 545) [34]. BF% estimated with this portable device has been validated against dual-energy X-ray absorptiometry [34]. Systolic and diastolic blood pressures were estimated in a seated position after a period of rest using a portable blood pressure monitor (the model used was an Omron HEM-705CP) on the upper arm.

Also at the time of the questionnaire administration, a small amount of capillary blood was collected via finger stick. Immediately after blood collection, hemoglobin (Hb) concentration was estimated with a hemoglobinometer (the model used was a HemoCue 201+) and glycated hemoglobin (HbA_1c_) percentage was estimated with a point of care test (A1C NOW manufactured by PTS Diagnostics). Additional blood specimen was allowed to fall freely on filter paper for dried blood spots (DBS). DBS were allowed to dry at ambient temperature for up to 24 h and were then stored frozen (− 20 °C) until assay at the icddr,b Immunobiology, Nutrition, and Toxicology Laboratory. DBS were evaluated for soluble transferrin receptor (sTfR), a biomarker of iron deficiency, by enzyme immunoassay (Ramco TFC-94) modified for use with DBS. One 3.2 mm disc of DBS specimen, equivalent to 1.5 μl serum, was removed with a hole punch and eluted in assay buffer overnight. Eluent was assayed without further dilution. For the 35 plates of sTfR included in these analyses, the intra-assay coefficient of variation (CV) was 3.48%, and the inter-assay CV was 14.8% at low concentration and 9.5% at high concentration.

For analyses, we used population-specific cut points to define chronic disease outcomes, when such cut points have been recommended (for obesity and type 2 diabetes) [35]. Obesity was defined using the World Health Organization recommended body mass index (BMI) cut off of > 24.9 kg/m^2^ for Asian populations [36]; high waist circumference was defined as > 35 in [37].; type 2 diabetes was defined as HbA1c ≥ 6.0% (the cut off for diabetes in Bangladeshi populations) or if the respondent reported having had a diabetes diagnosis [38]; high BF% was defined as > 35% [39]; hypertension was defined as systolic blood pressure ≥ 130 mmHg or diastolic blood pressure ≥ 80 mmHg [40]; iron deficiency was defined as TfR > 5 mg/l [41]; and, anemia was defined as hemoglobin < 12 g/dL [42].

### Husband migration

Two variables for husband migration were assessed: (1) a binary (ever/never) variable for any form of husband labor migration past or present, which included all participating women and captured broader trends; and (2) a binary variable indicating that a participant’s husband was a current international labor migrant compared with never having been a migrant, to capture the most dramatic contrast (women whose husbands were past migrants or domestic migrants were not included in this analysis). We used husband’s reported current location and remittances for this variable, with current international labor migration defined as husband in an international location or the combination of remittances of greater than or equal to 10,000 taka monthly and spouse reported as a migrant (for spouses visiting at home at the time of the survey).

### Statistical analysis

Outcomes of interest were underweight, obese, high BF%, waist circumference > 35 in., anemia, iron deficiency, hypertension, and diabetes. Logistic regression was used to estimate crude odds ratios (OR) and OR adjusted for age, education, religion, and betelnut use. Next, possible mediators (income, food security, and purchasing all food from bazaar) were added to logistic regression models. The effects of mediator variables that were statistically significant at an alpha level of 0.05 in the final logistic regression models were examined by estimating path coefficients via structural equation modeling (SEM). All data were analyzed in SAS 9.4.

### Qualitative and ethnographic methods

We supplemented interpretation of survey and health data with analysis of semi-structured interviews and participant observation. 48 semi-structured interviews of women in Matlab were conducted in the 2010 wave of the project; women were randomly sampled using the same inclusion criteria as the survey. Interviews consisted of 49 questions ranging from the interviewee’s personal health to changes in economic opportunities occurring in the community. All interviews were conducted in Bengali and later translated and transcribed into English by bilingual field team members. The translated interview transcripts were inductively coded using a grounded theory approach [43, 44]. Participant observation was conducted during 12 months of fieldwork by Shenk in Matlab, Bangladesh coinciding with two rounds of data collection in 2010 and 2017–2018; observations were made especially of variation in the living situations, daily work, occupation, and health of women in Matlab. Fieldnotes from participant observation were consulted in interpreting data from these analyses.

## Results

### Quantitative findings

Data were available from 621 currently married, non-pregnant women (Table 1). Both undernutrition and excess adiposity were common: 9.0% of participants were underweight, 50.9% were iron deficient, and 48.3% were anemic; while 39.6% of participants were obese, 27.3% had a waist circumference over 35 in., and 33.1% had a high BF%. Chronic disease was also common: 32.8% of participants were diabetic and 32.9% had hypertension. Nearly half of the women (44.9%) had a husband who never migrated. Of those whose husband had ever migrated, 72 (25.8%) had a husband who was a current international migrant. Among participants, the mean age was 47.7 years, the mean years of education was 5.2, the mean monthly household income was 17,453 taka, and 89.5% were Muslim. The majority of participants reported their main occupation as housewife (95.3%), nearly half used betelnut (48.5%), 67.6% reported always having enough food, and 59.5% reported purchasing all their food at the bazaar (as opposed to producing it themselves).

- Husband Ever Migrant

**Table 1:** Study Participants (*n* = 621)

	Mean (SD)/Frequency (%)
Age (years)	47.7 (11.6)
Education (years)	5.2 (4.4)
Religion is Islam	554 (89.5%)
Household income (Taka)	17,452.7 (19,407.6)
Main occupation is housewife	591 (95.3)
Betelnut use	301 (48.5%)
Food secure	417 (67.6%)
All food is from the bazaar	368 (59.5%)
Body fat 35% or more	202 (33.1%)
Anemia	300 (48.3%)
Waist over 35 in.	167 (27.3%)
Hypertension	202 (32.9%)
Diabetes (HbA_1C_ > =6.0)	192 (32.8%)
Iron deficiency (sTfR> 5 mg/l)	316 (50.9%)
Obese	242 (39.6%)
Underweight	56 (9.0%)
Husband was ever a labor migrant	279 (44.9%)
Husband is currently an international migrant	72 (11.6%)

Crude associations between husband migration (ever/never) and multiple chronic disease indicators and outcomes were apparent (Table 2, panel 1). After controlling for confounding variables, husband migration (ever) was positively associated with high BF% and waist circumference > 35 in. and inversely associated with anemia (Table 2, panel 2). Betelnut use was found to be associated with hypertension in Table 2, panel 2. Food security was negatively associated with underweight (Table 2, panel 3); income was positively associated with waist circumference > 35 in. and hypertension, and inversely associated with iron deficiency (Table 2, panel 3), while purchasing all food in the bazaar was associated with high BF% and obesity. Once income, food security, and purchasing all food from the bazaar were considered, betelnut use was no longer associated with hypertension (Table 2, panel 3).

**Table 2:** Logistic regression models assessing the association of chronic disease outcomes (OR, 95% CI) with a spouse who has ever migrated: crude associations (panel 1); adjusted for confounders (panel 2); and including possible mediators (panel 3) (n = 621)

Variable	Underweight	Obese	High Body Fat	High Waist Circ.	Anemia	Iron Deficiency	Hypertension	Diabetes
**Panel 1**								
Ever Migrant	0.66 (0.37, 1.17)	1.51 (1.09, 2.09)	1.42 (1.01, 1.99)	1.31 (0.92, 1.88)	0.63 (0.46, 0.86)	1.44 (1.05, 1.98)	0.85 (0.60, 1.18)	0.98 (0.69, 1.39)
**Panel 2**								
Ever Migrant	1.01 (0.54, 1.88)	1.15 (0.80, 1.65)	1.39 (0.96, 2.02)	1.43 (0.96, 2.12)	0.74 (0.52, 1.05)	1.22 (0.86, 1.73)	1.24 (0.84, 1.83)	1.21 (0.82, 1.79)
Age	1.02 (0.99, 1.05)	0.99 (0.97, 1.01)	1.02 (0.99, 1.04)	1.03 (1.01, 1.05)	1.02 (1.00, 1.04)	0.99 (0.97, 1.00)	1.05 (1.03, 1.07)	1.07 (1.05, 1.09)
Education	0.88 (0.81, 0.95)	1.06 (1.01, 1.09)	1.08 (1.04, 1.13)	1.04 (0.99, 1.09)	1.02 (0.98, 1.06)	1.05 (1.01, 1.09)	1.02 (0.98, 1.06)	1.02 (0.97, 1.06)
Islam	0.95 (0.38, 2.36)	1.23 (0.71, 2.14)	1.07 (0.60, 1.92)	0.90 (0.49, 1.65)	0.89 (0.52, 1.51)	1.38 (0.81, 2.34)	0.59 (0.34, 1.05)	0.94 (0.52, 1.71)
Betelnut use	1.13 (0.56, 2.24)	0.83 (0.54, 1.25)	1.11 (0.72, 1.72)	1.06 (0.67, 1.67)	1.28 (0.86, 1.91)	1.21 (0.81, 1.81)	1.61 (1.04, 2.48)	0.61 (0.39, 0.96)
**Panel 3**								
Ever Migrant	1.59 (0.78, 3.29)	1.01 (0.64, 1.59)	1.16 (0.72, 1.86)	1.29 (0.79, 2.11)	0.94 (0.61, 1.46)	1.42 (0.91, 2.20)	1.18 (0.73, 1.93)	0.98 (0.61, 1.58)
Age	1.01 (0.98, 1.05)	0.98 (0.96, 1.01)	1.02 (0.99, 1.05)	1.03 (1.00, 1.05)	1.02 (1.00, 1.05)	0.99 (0.97, 1.01)	1.06 (1.03, 1.09)	1.06 (1.04, 1.09)
Education	0.94 (0.85, 1.03)	1.03 (0.98, 1.09)	1.06 (1.00, 1.12)	1.00 (0.95, 1.06)	1.04 (0.99, 1.09)	1.05 (0.99, 1.10)	1.02 (0.96, 1.08)	1.04 (0.98, 1.09)
Islam	1.02 (0.29, 3.65)	1.59 (0.73, 3.43)	1.33 (0.59, 3.01)	0.67 (0.31, 1.44)	0.74 (0.36, 1.51)	1.39 (0.68, 2.85)	0.77 (0.35, 1.69)	1.24 (0.55, 2.77)
Betelnut use	1.13 (0.49, 2.58)	0.79 (0.48, 1.33)	1.11 (0.64, 1.90)	0.98 (0.56, 1.70)	1.29 (0.79, 2.13)	0.99 (0.59, 1.62)	1.54 (0.89, 2.64)	0.67 (0.38, 1.16)
Income	0.78 (0.55, 1.11)	1.10 (0.88, 1.39)	1.21 (0.95, 1.55)	1.31 (1.02, 1.69)	1.07 (0.86, 1.33)	0.78 (0.63, 0.98)	1.28 (1.00, 1.64)	0.99 (0.79, 1.27)
Food secure	0.37 (0.18, 0.74)	1.23 (0.76, 1.99)	1.22 (0.74, 2.03)	1.42 (0.84, 2.40)	0.89 (0.56, 1.40)	0.83 (0.53, 1.32)	0.92 (0.55, 1.52)	1.15 (0.69, 1.90)
All bazaar food	0.62 (0.31, 1.25)	1.89 (1.22, 2.94)	1.78 (1.12, 2.84)	1.54 (0.95, 2.48)	0.99 (0.65, 1.51)	1.34 (0.88, 2.04)	0.99 (0.62, 1.58)	1.17 (0.73, 1.86

**Fig. 1:**
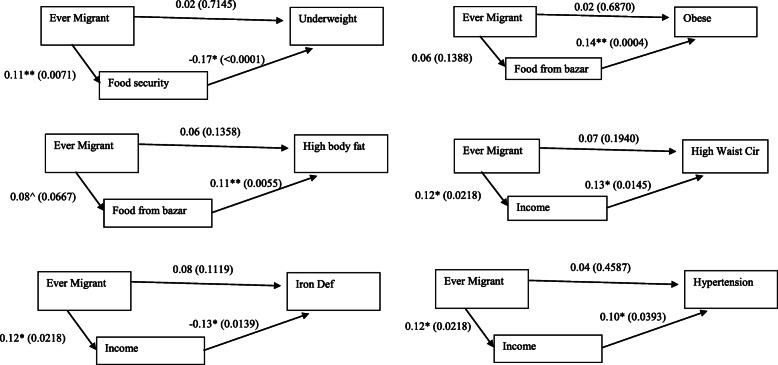
Path Analyses controlling for age, education, Islam, and betelnut use – Estimate (*p*-value)

Structural equation modeling showed that food security mediated an inverse association between husband migration (ever) and underweight, while income mediated an inverse association between husband migration (ever) and iron deficiency (Fig. 1). Income mediated a positive association between husband migration (ever) and waist circumference > 35 in, as well as the association with hypertension. Purchasing all food in the bazaar mediated the positive association between husband migration (ever) and obesity and high BF%. Supplementary Table 1 shows the direct pathway from having a husband who was ever a migrant to the chronic disease outcomes.

(b) Husband Current International Migrant

Patterns were similar when models were restricted to compare participants with current international migrant husbands with those with never migrant husbands. After controlling for confounding variables, a current international migrant husband was positively associated with high BF% (Table 3, panel 2). Food security was inversely associated with underweight, income was positively associated with multiple measures of adiposity and hypertension, while purchasing all food from the bazaar was positively associated with measures of adiposity (obesity and high BF%). Food security was also positively associated with high waist circumference.

**Table 3:** Logistic regression models assessing chronic disease outcomes (OR, 95% CI) with a spouse that is a current international migrant: crude associations (panel 1), adjusted for confounders (panel 2); and including possible mediators (panel 3) (*n* = 414)

Variable	Underweight	Obese	High Body Fat	High Waist Cir.	Anemia	Iron Def	Hypertension	Diabetes
**Panel 1**								
International Migrant	0.37 (0.11, 1.24)	1.32 (0.78, 2.21)	1.61 (0.95, 2.72)	0.79 (0.43, 1.48)	0.82 (0.49, 1.37)	1.59 (0.95, 2.66)	0.51 (0.28, 0.93)	0.60 (0.33, 1.10)
**Panel 2**								
International Migrant	0.83 (0.22, 3.18)	0.82 (0.45, 1.49)	1.69 (0.92, 3.14)	0.94 (0.46, 1.91)	1.49 (0.82, 2.68)	1.22 (0.68, 2.20)	1.04 (0.51, 2.11)	1.07 (0.53, 2.14)
Age	1.03 (0.99, 1.07)	0.99 (0.97, 1.02)	1.02 (0.99, 1.04)	1.03 (1.00, 1.05)	1.03 (1.01, 1.05)	0.99 (0.98, 1.02)	1.05 (1.02, 1.07)	1.07 (1.04, 1.09)
Education	0.91 (0.82, 0.99)	1.07 (1.02, 1.13)	1.08 (1.02, 1.14)	1.05 (0.99, 1.12)	0.99 (0.94, 1.04)	1.09 (1.03, 1.14)	1.00 (0.95, 1.06)	0.99 (0.94, 1.05)
Islam	0.89 (0.32, 2.46)	1.35 (0.70, 2.59)	0.96 (0.49, 1.86)	0.85 (0.42, 1.70)	0.61 (0.33, 1.15)	1.49 (0.79, 2.77)	0.49 (0.26, 0.93)	0.84 (0.43, 1.66)
Betelnut use	1.14 (0.49, 2.65)	0.73 (0.44, 1.22)	1.04 (0.60, 1.78)	0.99 (0.56, 1.76)	1.39 (0.85, 2.26)	1.16 (0.71, 1.89)	1.29 (0.76, 2.18)	0.55 (0.31, 0.97)
**Panel 3**								
International Migrant	2.05 (0.31, 13.43)	0.44 (0.18, 1.07)	0.96 (0.39, 2.33)	0.49 (0.18, 1.29)	3.43 (1.42, 8.29)	1.30 (0.56, 3.02)	0.57 (0.21, 1.55)	0.66 (0.26, 1.72)
Age	1.04 (0.99, 1.09)	0.98 (0.96, 1.02)	1.02 (0.99, 1.05)	1.02 (0.99, 1.06)	1.05 (1.02, 1.08)	1.00 (0.97, 1.03)	1.04 (1.01, 1.07)	1.05 (1.02, 1.09)
Education	0.99 (0.88, 1.12)	1.02 (0.95, 1.09)	1.04 (0.97, 1.12)	0.99 (0.92, 1.07)	1.04 (0.98, 1.11)	1.09 (1.02, 1.16)	0.98 (0.92, 1.06)	0.99 (0.93, 1.07)
Islam	0.97 (0.20, 4.66)	1.74 (0.69, 4.38)	0.98 (0.39, 2.46)	0.52 (0.21, 1.26)	0.43 (0.18, 1.04)	1.37 (0.59, 3.19)	0.59 (0.25, 1.41)	1.48 (0.57, 3.82)
Betelnut use	1.27 (0.42, 3.83)	0.70 (0.37, 1.34)	1.04 (0.52, 2.05)	0.88 (0.43, 1.82)	1.29 (0.69, 2.41)	0.89 (0.48, 1.65)	1.27 (0.65, 2.47)	0.76 (0.38, 1.52)
Income	0.78 (0.48, 1.26)	1.51 (1.08, 2.11)	1.54 (1.09, 2.17)	1.71 (1.18, 2.47)	0.84 (0.62, 1.13)	0.80 (0.59, 1.08)	1.37 (0.99, 1.90)	1.12 (0.82, 1.54)
Food secure	0.31 (0.12, 0.79)	1.54 (0.83, 2.86)	1.23 (0.65, 2.33)	2.04 (1.00, 4.14)	0.67 (0.37, 1.19)	0.73 (0.41, 1.29)	1.03 (0.56, 1.91)	1.53 (0.81, 2.89)
All bazaar food	0.48 (0.19, 1.21)	2.21 (1.24, 3.95)	1.82 (1.00, 3.32)	1.78 (0.94, 3.37)	0.65 (0.38, 1.13)	1.16 (0.68, 1.97)	1.13 (0.63, 2.02)	1.50 (0.83, 2.73)

**Fig. 2:**
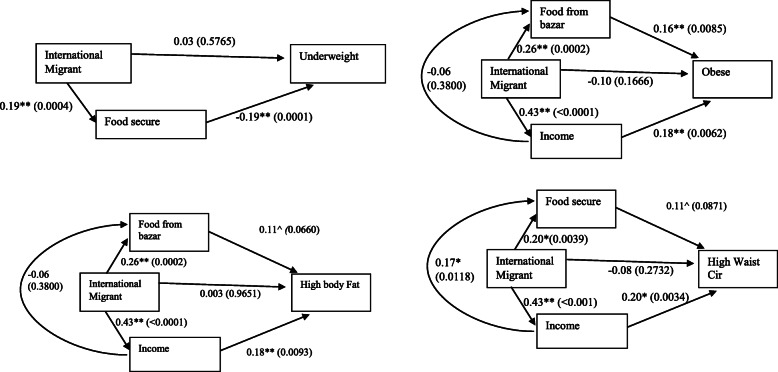
Path Analyses controlling for age, education, Islam, and betelnut use – Estimate (p-value)

Structural equation modeling showed that food security mediated an inverse association between husband’s international migration and underweight (Fig. 2). Income and food from the bazaar mediated associations between husband’s international migration and adiposity for both the obesity and high BF% outcomes. Income and food security mediated an association between an international migrant husband and high waist circumference. Supplementary Table 2 shows the direct pathway from having a husband who was currently an international migrant to the chronic disease outcomes.

### Qualitative findings

Complete interviews were available for 48 women ranging in age from 20 to 65 with a mean age of 42.3; 9 had husbands who were current labor migrants, while others had close relatives (husbands, fathers, sons, others) who either were currently or had been labor migrants in the past. Most women discussed labor migration in a positive light as a phenomenon with clear economic benefits for individuals. Of the 48 interviews coded, 41 women said positive things about labor migration while mentioning no negatives, while only one woman mentioned both pros and cons. Of the 42 women who expressed positive views on labor migration, 28 focused on its favorable economic consequences. For example, one 49-year-old woman expressed: “*There are good effects from the people leaving the village for work. Now people work abroad or in the city and earn money and fulfill their dreams. They build new brick houses instead of bamboo-made houses, buy more land for cultivation, or spend money for their daughter’s marriage. But I see no bad effects from people leaving the village*.” Similarly, from a 20-year-old woman whose husband was currently working in Saudi Arabia: “*There are good effects on people for leaving the village for work. Today people earn lots of money and buy land. Build new houses. Now they can eat good food and wear good clothes and try to change their way of living. If someone has enough money then people give him respect*.”

Women expressed concerns about poor health, with 60% reporting concerns related to chronic illnesses including heart disease, blood pressure, strokes, diabetes, and cancer, while 40% raised concerns related to infectious disease or other health issues. Women also commonly expressed ideas consistent with the nutrition and epidemiologic transitions; for example one 39-year-old woman perceived: “*Now people are dying less. In the past children were dying more but now adult people aged 25, 30, 40 are dying more. In the past people were dying for want of food and treatment. Now people are dying for blood pressure.*” While women do not mention obesity as a specific concern, two sets of concerns raised are consistent with the quantitative results. First, the focus on chronic diseases such as heart disease and diabetes, for which obesity is a risk factor, comes across clearly in the transcripts. Second, many women share concerns focusing on changes in the food system and the connection between food and chronic illness. For example, a 34-year-old respondent said, “*Now diseases are more. Why it is more I don’t know. People are taking good food (nutritious food) but still they are sick*.”

Our ethnographic observations point to clear links between incomes, diets, and physical activity in this rural setting. Rice is the staple food, with most people eating rice with 2–3 meals per day. Poorer people have less rice available, but also less varied diets with more limited access to meat or fish. Increased income typically allows people to eat more meat and fish and a wider variety of vegetables; it also allows the more frequent purchase of sodas and snack foods. If family members are personally engaged in agriculture, animal husbandry, or fishing, however, individuals at any income level may have greater food security and/or greater diet breadth (most commonly through vegetables, fish, or eggs) due to their direct access to food production. Poorer women typically engage actively in physical labor, sometimes performing agricultural tasks, but are virtually always actively responsible for housework: cooking full meals 2–3 times a day, washing clothes and dishes by hand, cleaning the house, and caring for children. Wealthier women, in contrast, rarely work in the fields or perform other agriculture-related tasks, and often pay others to help with the heavier parts of household labor, thus their levels of physical activity are generally lower.

## Discussion

The nutrition transition that has been reported for populations throughout South Asia was apparent in our study community: overweight, obesity, and chronic diseases were quite common, with around one third of participating women exhibiting obesity, high BF%, high waist circumference, hypertension, and/or diabetes. Whether this represents a true transition to low rates of undernutrition and infectious disease mortality remains to be seen, as we also observed high rates of underweight, iron deficiency, and anemia. These findings are consistent with patterns of dual burden malnutrition—the combination of high rates of both undernutrition and overnutrition—described in South Asia and elsewhere in the world and thought to accompany transitional stages of the epidemiologic and nutrition transitions [45]. It is not clear whether all countries will inevitably experience the same stages of the nutrition transition. What is clear is that greater access to processed foods and more sedentary lifestyles are increasing in low and middle income countries, which are known risk factors for chronic disease [46].

Our findings suggest that out-migration of men, particularly internationally, contributes to both lower risk of undernutrition (anemia and underweight) and higher risk for excess adiposity (obesity, high BF%, and high waist circumference) among wives who remain in the rural village. This may be due to higher incomes among those receiving remittances from migrants, which our qualitative data suggest improve access to bazaar-purchased food. Greater reliance on purchased foods, rather than foods produced within a household (in farms, gardens, or fish ponds), implies paradoxically both improvement and decline in dietary quality. While gains in income often are associated with increased food security and lead families to purchase more and higher-quality foods such as additional vegetables, fruit, meat and fish from bazaars, over time and with additional income, interviews and observations suggest that they also lead to increases in the purchase of processed foods high in added sugars (such as soda, snack foods, and sweets). In addition, the transition to more bazaar-purchased foods is associated with a lifestyle change away from farming and gardening, which, for women, likely involves less physical activity [22]. In Matlab, not only do women from higher income households do less agricultural work, but they also have reduced levels of activity related to housework because women in the rising middle class routinely pay for household help to aid in domestic tasks.

Consistent with our qualitative finding that remittance incomes can dramatically change the diets of wives of labor migrants, our structural equation models indicated that protective effects of husband’s labor migration against undernutrition were generally mediated by higher food security and income. Similarly, increased risk for excess adiposity among labor migrants’ wives was generally mediated by purchasing food at the bazaar, along with income. Here, income likely captures both increased purchasing power for foods and lower physical activity levels.

These findings accord with other research documenting positive associations between higher SES and overweight/obesity in Bangladesh, as well as in other low and middle income countries [47, 48]. This is underlined by data from 37 countries clearly showing that most low and middle income countries (including Bangladesh) had lower levels of overweight in the lower wealth group [49]. By contrast, in high-income countries, obesity risk is generally highest among those with low SES [50]. This contrast is consistent with the nutrition transition theory, in that the final stages of the nutrition transition are posited to include a shift from diets high in fat and sugar alongside a sedentary lifestyle (e.g. what is emerging among those with rising incomes and middle class lifestyles in Bangladesh) toward improved diets and increased physically activity (e.g. higher income people in high-income countries) [3]. This contrast also likely reflects complexity in the etiology of obesity, which is influenced by both diet and stress, particularly during early life [51–53]. In low and middle income countries in general, and in Bangladesh in particular, it is likely that the majority of adults—particularly those from rural areas and relatively low-income backgrounds, including women in Matlab—experienced nutritional stress in early life, before the improvements in recent decades including food distribution, stabilization of grain markets, and other factors decreased risk for famine [54]. Such early nutritional stress may result in lasting increases in risk for chronic disease among those who are later exposed to obesogenic and diabetogenic diets [55, 56]. Furthermore, accumulating evidence suggests that there exists substantial intergenerational risk for obesity and type 2 diabetes, as the epigenetic changes that connect early nutritional stress to disordered metabolism and obesity affect uterine physiology, and thus the in utero nutrition available to the next generation [57–59].

The key limitation of this study was its cross-sectional design, which is vulnerable to reverse causation (e.g., women prone to excess adiposity may be more likely to marry a man in a good position to be an international labor migrant or the families of international labor migrants may have high household incomes prior to sending their husband or son abroad to work). Additionally, as these data were collected in rural Bangladesh, they may have limited generalizability in other settings. Yet the consistency of these results with other findings from the region and elsewhere in the world—alongside our qualitative findings—support the likelihood of the pathways described here from increasing labor migration to both positive and negative health outcomes.

## Conclusions

Our findings implicate labor migration, and particularly international labor migration, in intensifying the nutrition transition in Bangladesh by both decreasing left-behind wives’ risk for undernutrition and increasing their risk for excess adiposity. Further, our findings show that both higher incomes and greater reliance on purchased (rather than produced) foods mediate the effect of husband’s labor migration on chronic disease indicators. Reliance on purchased foods implies increased dietary intake of added sugars, while higher incomes imply decreased physical activity, both of which may provide targets for development of interventions to prevent chronic diseases like obesity among adult women in rural areas throughout South Asia.

## Supplementary Information

**Additional file 1.**

## Data Availability

The dataset analyzed during the current study is not publicly available due to ethical review board restrictions but is available from the corresponding author on reasonable request.
