# Supplementary material for: Labor migration is associated with lower rates of underweight and higher rates of obesity among left-behind wives in rural Bangladesh: a cross-sectional study
Source: Global Health. 2021 Jul 18;17:81. doi: 10.1186/s12992-021-00712-5 (PMC8286616; doi:10.1186/s12992-021-00712-5)
Supplement: Supplementary file 1 — Additional file 1. [file 12992_2021_712_MOESM1_ESM.docx]

| Supplemental Table 1. Direct estimates controlling for age, education, religion, and betelnut use (p-value) | | | | | | | | |
| --- | --- | --- | --- | --- | --- | --- | --- | --- |
| Variable | Underweight | Obese | High Body Fat | High Waist Circ. | Anemia | Iron Deficiency | Hypertension | Diabetes |
| Ever Migrant | -0.001 (0.9672) | 0.03 (0.4403) | 0.07 (0.0855) | 0.08 (0.0767) | NA | 0.05 (0.2621) | 0.05 (0.2695) | NA |

| Supplemental Table 2. Direct estimates controlling for age, education, religion, and betelnut use (p-value) | | | | | | | | |
| --- | --- | --- | --- | --- | --- | --- | --- | --- |
| Variable | Underweight | Obese | High Body Fat | High Waist Cir. | Anemia | Iron Def | Hypertension | Diabetes |
| International Migrant | -0.006 (0.9186) | -0.04 (0.5146) | 0.09 (0.0.0938) | -0.01 (0.8382) | NA | NA | NA | NA |
